# Association between the triglyceride–glucose index and mortality in critically ill patients: A meta-analysis

**DOI:** 10.1097/MD.0000000000039262

**Published:** 2024-08-16

**Authors:** Mengzhen Nie, Biantong Jiang, Yu Xu

**Affiliations:** a Department of Critical Care Medicine, West China Hospital/West China School of Nursing, Sichuan University, Chengdu, China.

**Keywords:** critically ill, diabetes mellitus, intensive care unit, mortality, triglyceride–glucose index

## Abstract

**Background:**

To further identify the association of the triglyceride–glucose (TyG) index with the risk of mortality among critically ill patients admitted to the intensive care unit (ICU).

**Methods:**

The PubMed, Web of Science, and EMBASE databases were searched for relevant studies up to February 2, 2024. The primary outcomes were in-hospital mortality and ICU mortality. The secondary outcomes were 30-day mortality, 90-day mortality, and 1-year mortality. The hazard ratios (HRs) with 95% confidence intervals (CIs) were combined to evaluate the associations between the TyG index and the above endpoints. All the statistical analyses were performed with STATA 15.0 software.

**Results:**

Ten studies involving 22,694 patients were included. The pooled results demonstrated that an elevated TyG index indicated an increased risk of in-hospital mortality (HR = 1.76, 95% CI: 1.41–2.18, *P* < .001), ICU mortality (HR = 1.52, 95% CI: 1.33–1.74, *P* < .001), 30-day mortality (HR = 1.50, 95% CI: 1.02–2.19, *P* = .037), 90-day mortality (HR = 1.42, 95% CI: 1.01–2.00, *P* = .043), and 1-year mortality (HR = 1.19, 95% CI: 1.11–1.28, *P* < .001). Subgroup analysis for in-hospital mortality and ICU mortality based on sex, age, body mass index and hypertension showed similar results. However, subgroup analysis stratified by diabetes mellitus (DM) revealed that the associations of the TyG index with in-hospital mortality (HR = 2.21, 95% CI: 1.30–3.78, *P* = .004) and ICU mortality (HR = 1.93, 95% CI: 0.95–3.94, *P* = .070) were observed only among patients without DM.

**Conclusion:**

The TyG index was significantly associated with mortality among critically ill patients without DM, and an elevated TyG index predicted an increased risk of mortality.

## 1. Introduction

The characteristics of critically ill patients include prolonged hospital stays, high mortality rates, and significant family burdens.^[1]^ These patients often experience multiple diseases, severe internal environment disruptions, complex metabolic imbalances, and challenging, rapidly changing, and unpredictable medical conditions. Effectively assessing the severity and prognosis of patients and accurately specifying treatment plans remain continuous high-priority issues that demand close attention.^[2,3]^ According to data released by the World Health Organization in 2019, the top 6 causes of death worldwide are hypertension, unhealthy diet, high blood sugar, air pollution exposure, high body mass index (BMI), and hyperlipidemia, among others. It is evident that a significant portion of these primary causes of death and risk factors are closely associated with age and metabolic abnormalities.^[4]^

Insulin resistance is a common pathophysiological change observed in critically ill patients, and it is associated with the progression of the disease.^[5]^ Insulin resistance is defined as a condition where there is a decrease in the efficiency of insulin in promoting glucose uptake and utilization, and it is a prominent characteristic of metabolic syndrome.^[5]^ The triglyceride–glucose (TyG) index, calculated from fasting triglyceride and fasting blood glucose levels, is a widely recognized novel marker and is considered a simple and reliable surrogate for insulin resistance.^[6]^ Previous research on the TyG index has mostly focused on chronic disease patients, for whom the TyG index has been reported to be an effective indicator for predicting the risk of mortality from various chronic illnesses.^[7–10]^ However, the prognostic value of the TyG index for critically ill patients has not yet been clearly established.

Therefore, the aim of this meta-analysis was to further elucidate the association between the TyG index and the risk of mortality among critically ill patients admitted to the ICU using currently available evidence, which might be beneficial for the clinical management and improvement of prognosis in critically ill patients.

## 2. Materials and methods

This current meta-analysis was conducted according to the Preferred Reporting Items for Systematic Review and Meta-Analyses 2020.^[11]^

### 2.1. Literature search

The PubMed, EMBASE, and Web of Science databases were searched from database inception to February 2, 2024, with the following terms: triglyceride glucose index, TyG, intensive care unit, ICU, mortality and death. The detailed search strategy used for the PubMed database is presented in Table, Supplemental Digital Content, http://links.lww.com/MD/N348. MeSH terms and free texts were applied, and references cited in the included studies were reviewed.

### 2.2. Inclusion criteria

Studies that met the following criteria were included: included patients who were critically ill and admitted to the ICU; the TyG index was calculated based on the levels of blood triglycerides and glucose at admission to the ICU; the associations between the TyG index and mortality, including in-hospital mortality, ICU mortality, 30-day mortality, 90-day mortality, and 1-year mortality, were evaluated; hazard ratios (HRs) with 95% confidence intervals (CIs) were reported; and full texts were available.

### 2.3. Exclusion criteria

Studies that met the following criteria were excluded: had duplicated or severely overlapping data or were reviews, case reports, editorials, animal trials, meeting abstracts, or letters.

### 2.4. Data retraction

The following information was collected from the included studies: the name of the first author, publication year, country, source of database, sample size, disease type, age, threshold of the TyG index, endpoint, and HR with the corresponding 95% CIs.

### 2.5. Methodological quality evaluation

The methodological quality was evaluated by the Newcastle–Ottawa scale (NOS) score, and studies with NOS scores≥6 were defined as high-quality studies.^[12]^

In our meta-analysis, the literature search, selection, data retraction, and quality assessment were performed by 2 authors, and any disagreements were resolved by team discussion.

### 2.6. Statistical analysis

The heterogeneity between studies was calculated by *I*^2^ statistics and the *Q* test. If significant heterogeneity was detected (indicated by *I*^2^ > 50% and/or *P* < .1), the random-effects model was applied; otherwise, the fixed-effects model was applied.^[13]^ HRs and 95% CIs were combined to evaluate the relationship between the TyG index and mortality. Subgroup analysis based on sex, age, BMI, history of diabetes mellitus (DM), and hypertension status was performed. Sensitivity analysis was performed to detect the sources of heterogeneity and assess the reliability of the pooled results. In addition, Begg funnel plot and Egger test were conducted to detect publication bias, and significant publication bias was defined as *P* < .05.^[14,15]^ If obvious publication bias was detected, then the fill-and-trim method was applied to identify potentially unpublished studies.^[16]^ The analysis was performed with STATA version 15.0 software (StataCorp LLC, College Station).

## 3. Results

### 3.1. Literature search and selection

**Figure 1.**
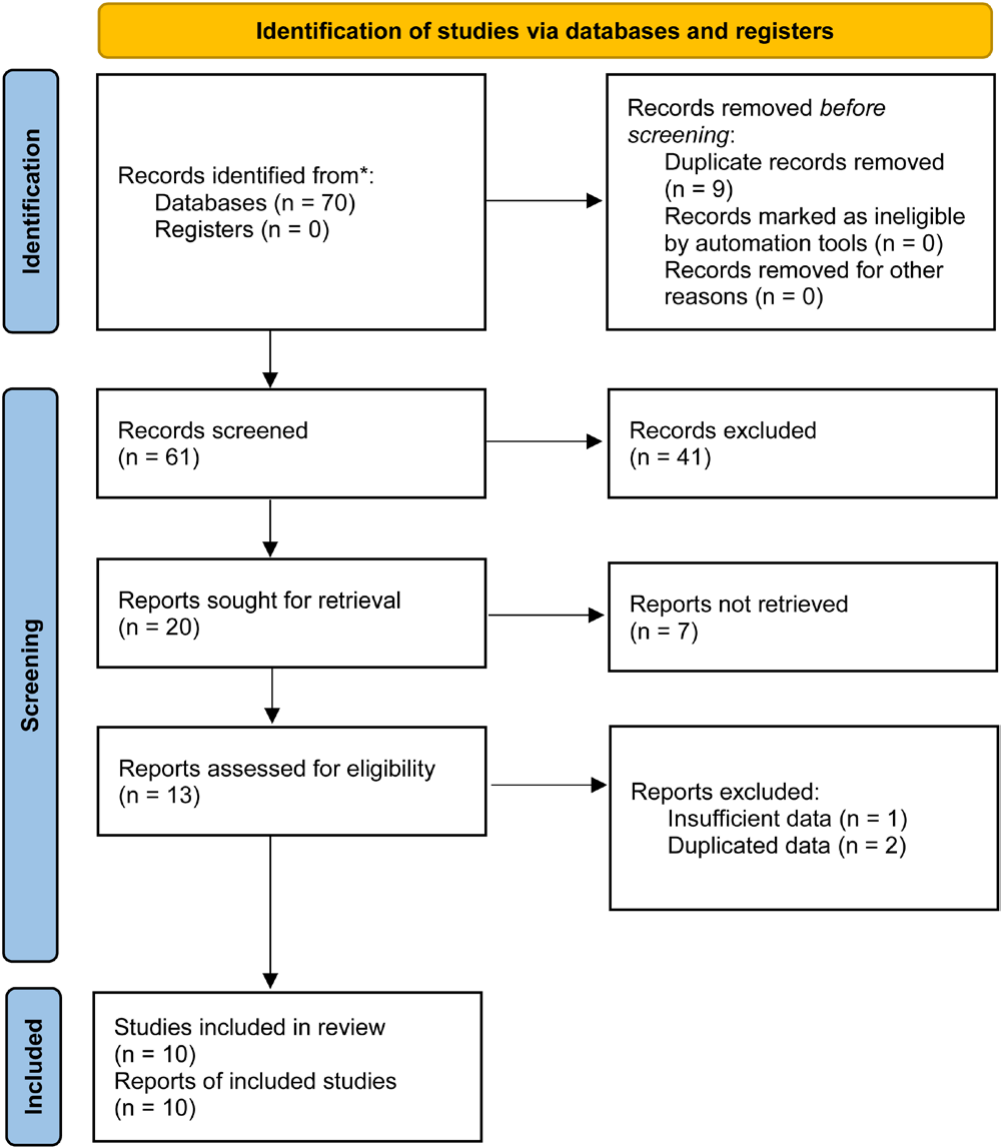
The PRISMA flow diagram of this meta-analysis. PRISMA = Preferred Reporting Items for Systematic Review and Meta-Analyses.

As shown in Figure 1, 70 records were identified from the 3 electronic databases. After reviewing the titles, abstracts, and full texts, 10 relevant studies were eventually included in our meta-analysis.^[17–26]^

### 3.2. Basic characteristics of the included studies

All included studies were retrospective, and a total of 22,694 patients were enrolled, with sample sizes ranging from 537 to 4839. Notably, in the study by Chen et al, 2 groups of patients were separately analyzed. Most patients were from the Medical Information Mart for Intensive Care III or IV database. In addition, all studies were high-quality studies with NOS scores of 6 or higher (Table 1).

**Table 1:** Basic characteristics of included studies.

Author	Year	Country (database)	Sample size	Diseases	Age (yr)	Threshold of TyG index	Subgroup analysis	Endpoints	NOS
Zhang^[17]^	2020	USA (eICU Collaborative Research Database)	4570	Stroke	66.3 ± 14.2	Continuous	None	ICU mortality, hospital mortality	7
Liu^[18]^	2021	USA (MIMIC-III database)	1298	Subarachnoid hemorrhage	57.64 ± 19.79	BG: 155.33 mg/dL and BG: 8.65 mmol/L	None	30-day mortality, 90-day mortality	7
Liao^[19]^	2022	USA (MIMIC-III database)	3026	NR	65.44 ± 16.07	Continuous	Sex, age, BMI, DM, hypertension	ICU mortality, hospital mortality	7
Zhai^[20]^	2022	USA (eICU Collaborative Research Database)	4839	Heart disease	65.2 ± 13.8	Continuous	Sex, age, BMI, DM, hypertension, hypercholesterolemia	Hospital mortality	8
Boshen^[21]^	2023	USA (eICU Collaborative Research Database)	1021	Cardiac arrest	65 (54–76)	9.2	None	ICU mortality, hospital mortality	8
Cai^[22]^	2023	USA (MIMIC-IV database)	733	Ischemic stroke	69 (58–79)	Continuous	Sex, age, BMI, DM	ICU mortality, hospital mortality	7
Chen^[23]^	2023	USA (MIMIC-IV database)	537	Cerebral hemorrhage	71 (median)	9.239	None	Hospital mortality	6
Chen^[23]^	2023	USA (MIMIC-IV database)	872	Cerebral infarction		9.251	None	Hospital mortality	6
Dai^[24]^	2023	USA (MIMIC-III database)	3902	NR	63.1 ± 15.9	Continuous	Sex, age	1-year mortality	8
Ye^[25]^	2023	USA (MIMIC-IV database)	639	Chronic kidney disease with coronary artery disease	75 (66–83)	Continuous	Sex, age	Hospital mortality, 1-year mortality	6
Zheng^[26]^	2023	USA (MIMIC-IV database)	1257	Sepsis	62.49 ± 17.27	Continuous	Sex, age, BMI, hypertension	ICU mortality, hospital mortality	7

BG = blood glucose, BMI = body mass index, DM = diabetes mellitus, ICU = intensive care unit, MIMIC = Medical Information Mart for Intensive Care, NOS = Newcastle–Ottawa Scale, NR = not reported, TyG = triglyceride–glucose index.

### 3.3. Association between the TyG index and in-hospital mortality among critically ill patients

**Figure 2.**
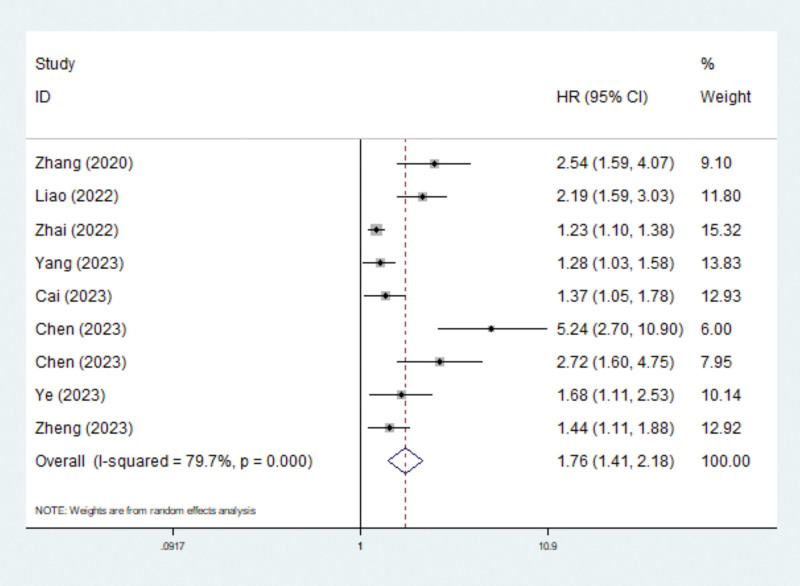
Association between TyG index and in-hospital mortality in critically ill patients. TyG = triglyceride glucose.

Eight studies explored the prognostic role of the TyG index for in-hospital mortality.^[17,19–23,25,26]^ The pooled results demonstrated that the TyG index was significantly related to in-hospital mortality among patients admitted to the ICU (HR = 1.76, 95% CI: 1.41–2.18, *P* < .001; *I*^2^ = 79.7%, *P* < .001) (Fig. 2). Subgroup analysis based on sex (male vs female), age (advanced age vs young age), BMI (high BMI vs normal or low BMI), hypertension (combined with hypertension vs without hypertension), and DM (combined with DM) revealed similar results (Table 2). However, among patients with DM, the TyG index was not significantly related to in-hospital mortality (HR = 1.21, 95% CI: 0.93–1.56, *P* = .151; *I*^2^ = 0.0%, *P* = .884) (Table 2).

**Table 2:** Results of meta-analysis.

	No. of studies	Hazard ratio	95% confidence interval	*P* value	*I*^2^ (%)	*P* value for heterogeneity
In-hospital mortality	8	1.76	1.41–2.18	<.001	79.7	<.001
Sex						
Male	5	1.58	1.19–2.11	.002	66.2	.018
Female	5	1.73	1.37–2.19	<.001	60.4	.039
Age						
Advanced age	5	1.91	1.30–2.81	.001	81.2	<.001
Young age	5	1.83	1.50–2.24	<.001	0.0	.714
BMI						
High BMI	4	1.43	1.17–1.75	<.001	42.9	.154
Normal or low BMI	4	1.71	1.15–2.55	.007	79.7	.002
DM						
Combined with DM	3	1.21	0.93–1.56	.151	0.0	.884
Without DM	3	2.21	1.30–3.78	.004	90.2	<.001
Hypertension						
Combined with hypertension	3	1.50	0.94–2.37	.086	65.2	.057
Without hypertension	3	2.01	1.68–2.41	<.001	43.2	.172
ICU mortality	5	1.52	1.33–1.74	<.001	35.7	.184
Sex						
Male	3	1.28	1.09–1.51	.003	0.0	.424
Female	3	1.64	1.22–2.22	.001	58.0	.093
Age						
Advanced age	3	1.30	1.10–1.53	.002	33.7	.221
Young age	3	1.77	1.43–2.20	<.001	48.3	.145
BMI						
High BMI	3	1.30	1.06–1.60	.013	0.0	.442
Normal or low BMI	3	1.30	1.10–1.54	.002	43.9	.168
DM						
Combined with DM	2	1.02	0.81–1.29	.860	0.0	.586
Without DM	2	1.93	0.95–3.94	.070	91.1	.001
Hypertension						
Combined with hypertension	2	1.27	0.78–2.06	.333	56.1	.131
Without hypertension	2	2.04	1.59–2.61	<.001	0.0	.984
30-day mortality	1	1.50	1.02–2.19	.037	-	-
90-day mortality	1	1.42	1.01–2.00	.043	-	-
1-year mortality	2	1.19	1.11–1.28	<.001	0.0	.375

BMI = body mass index, DM = diabetes mellitus, ICU = intensive care unit.

### 3.4. Association between the TyG index and ICU mortality among critically ill patients

**Figure 3.**
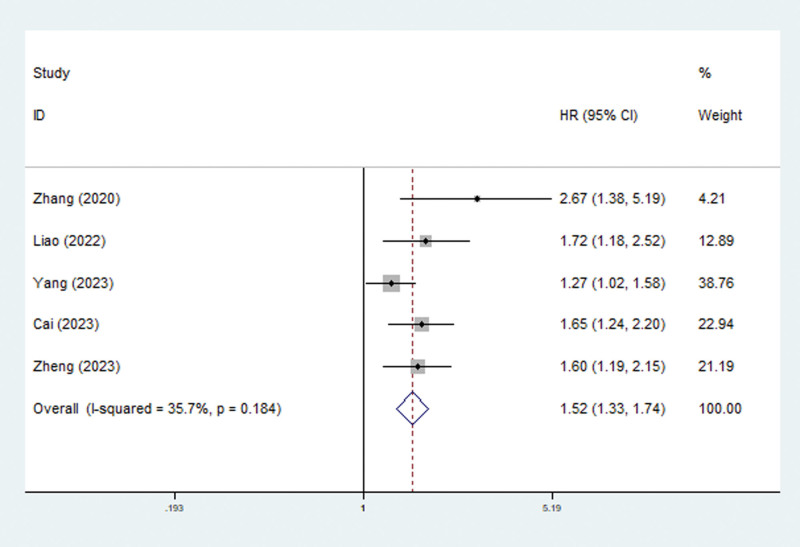
Association between TyG index and ICU mortality in critically ill patients. ICU = intensive care unit, TyG = triglyceride glucose.

Five studies explored the association between the TyG index and ICU mortality.^[17,19,21,22,26]^ The pooled results revealed that an elevated TyG index predicted an increased risk of ICU mortality (HR = 1.52, 95% CI: 1.33–1.74, *P* < .001; *I*^2^ = 35.7%, *P* = .184) (Fig. 3). Subgroup analysis stratified by sex (male vs female), age (advanced age vs young age), BMI (high BMI vs normal or low BMI), hypertension (combined with hypertension vs without hypertension), and DM (combined with DM) yielded similar results (Table 2). However, in patients with DM, the TyG index was not related to ICU mortality (HR = 1.02, 95% CI: 0.81–1.29, *P* = .860; *I*^2^ = 0.0%, *P* = .586) (Table 2).

### 3.5. Associations between the TyG index and 30-day, 90-day, and 1-year mortality among critically ill patients

**Figure 4.**
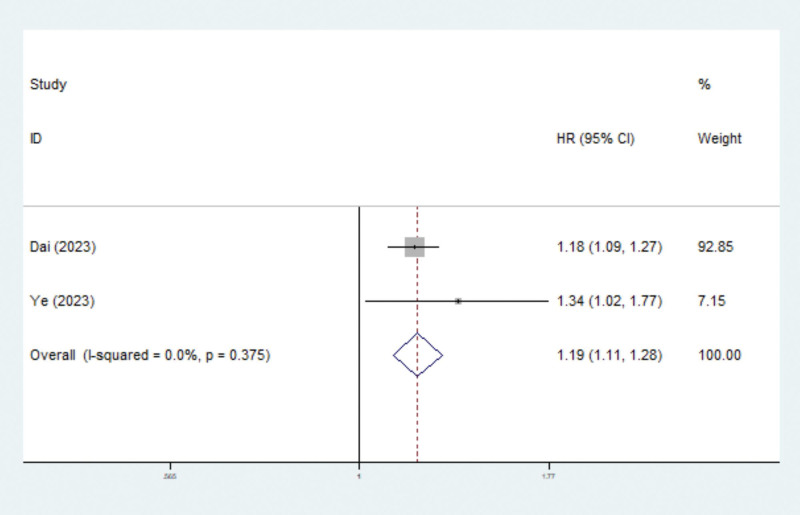
Association between TyG index and 1-year mortality in critically ill patients. TyG = triglyceride glucose.

According to the study by Liu et al, the TyG index was significantly associated with the risk of 30-day (HR = 1.50, 95% CI: 1.02–2.19, *P* = .037) and 90-day (HR = 1.42, 95% CI: 1.01–2.00, *P* = .043) mortality. In addition, 2 studies explored the relationship between the TyG index and 1-year mortality,^[24,25]^ and pooled results indicated that an elevated TyG index predicted an increased risk of 1-year mortality (HR = 1.19, 95% CI: 1.11–1.28, *P* < .001) (Fig. 4; Table 2).

### 3.6. Sensitivity analysis for the association between the TyG index and in-hospital mortality among critically ill patients

**Figure 5.**
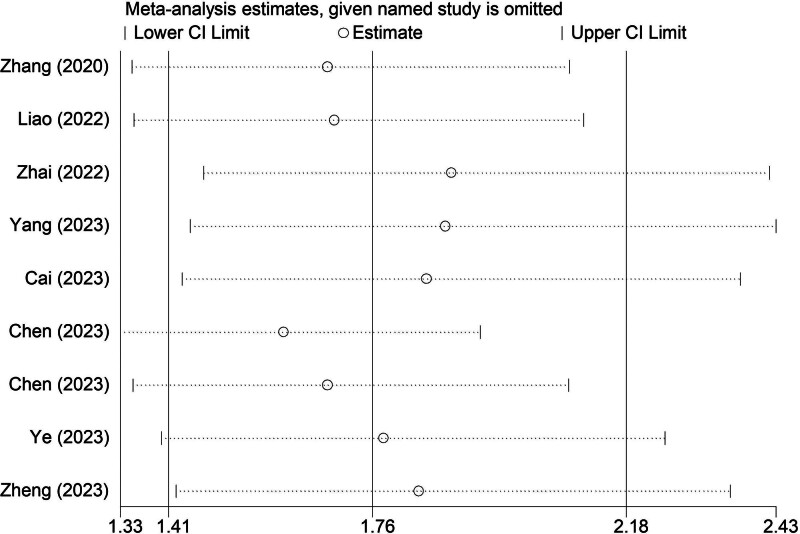
Sensitivity analysis for the association between TyG index and in-hospital mortality in critically ill patients. TyG = triglyceride glucose.

We further conducted a sensitivity analysis for in-hospital mortality, which indicated that our results were stable and reliable and that none of the included studies had a significant impact on the overall conclusion (Fig. 5).

### 3.7. Publication bias for the association between the TyG index and in-hospital mortality among critically ill patients

**Figure 6.**
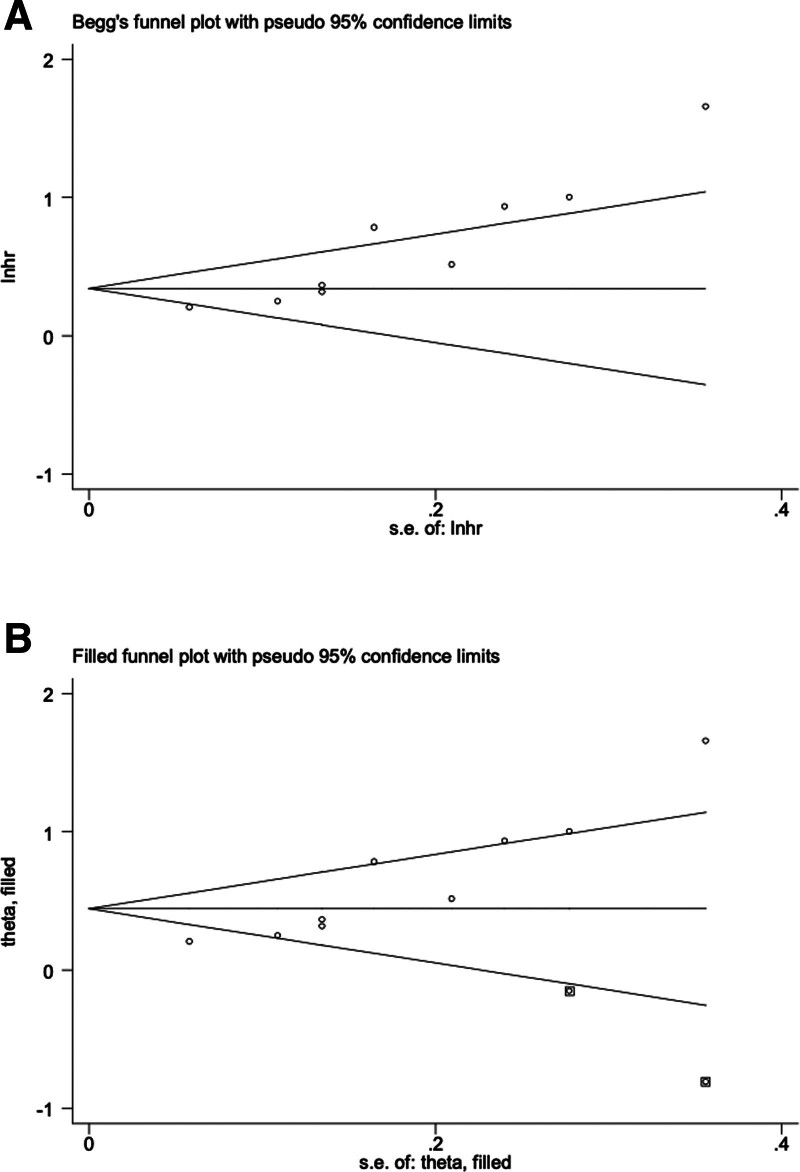
Begg (A) and filled (B) funnel plots for the association between TyG index and in-hospital mortality in critically ill patients. TyG = triglyceride glucose.

According to Begg funnel plot (Fig. 6A) and Egger test (*P* = .001) results, obvious publication bias was detected. Therefore, the fill-and-trim method was applied, and 2 potentially “unpublished” studies were identified (Fig. 6B). However, after combining these 2 studies, the results remained stable (fixed: HR = 1.38, 95% CI: 1.27–1.49, *P* < .001; random: HR = 1.56, 95% CI: 1.25–1.95, *P* < .001), which indicated that these potentially “unpublished” studies did not have a significant impact on the overall conclusion.

## 4. Discussion

The current meta-analysis included 10 available studies and demonstrated that the TyG index was significantly associated with mortality among critically ill patients who were admitted to the ICU. Patients with an elevated TyG index experienced an increased risk of all-cause mortality. However, subgroup analysis further revealed a significant association between the TyG index and mortality only among non-DM patients. Therefore, the TyG index could serve as a novel and reliable prognostic indicator among critically ill patients without DM based on our results.

Several meta-analyses have explored the prognostic value of the TyG index in patients with different diseases. Kohar et al included 31,671 postpercutaneous coronary intervention patients with acute coronary syndrome and reported that a higher TyG index was strongly related to major adverse cardiovascular events.^[27]^ In addition, Azarboo et al enrolled 6171 participants from 9 studies and demonstrated that the TyG index was significantly associated with the incidence of atrial fibrillation.^[28]^ Furthermore, Behnoush et al conducted a meta-analysis including 16,026 individuals and revealed that the TyG index was correlated with the occurrence of obstructive sleep apnea.^[29]^ However, the association between the TyG index and mortality in critically ill patients has not yet been verified by a meta-analysis.

Through various physiological and pathological processes, insulin resistance contributes to an increased risk of mortality in critically ill patients. Insulin resistance status is closely associated with chronic inflammation markers.^[30]^ In critically ill patients, the body may be in a state of overactive inflammation, leading to immune system dysfunction.^[30]^ These inflammatory and immune response can exacerbate organ damage, increasing the risk of death.^[31,32]^ In addition, insulin resistance results in a diminished response of the body to insulin, reducing cellular uptake and utilization of glucose. This may lead to elevated blood glucose levels and metabolic disturbances, increasing the risk of multiorgan dysfunction and, consequently, increasing the risk of death.^[33]^ Furthermore, insulin resistance is linked to an increased risk of cardiovascular diseases.^[34]^ In critically ill patients, cardiovascular complications such as myocardial infarction and heart failure may be more likely to occur, further increasing the likelihood of death.^[35,36]^ In addition, insulin resistance may cause abnormalities in coagulation and microcirculation, increasing the risk of thrombosis.^[37]^ In critically ill patients, this can lead to vascular occlusion, affecting organ perfusion and exacerbating the severity of the condition.^[37]^ Insulin resistance can adversely affect the function of multiple organs, including the liver, kidneys, and pancreas. This may result in organ dysfunction, increasing the susceptibility of critically ill patients to multiple organ failure.^[38]^ Therefore, insulin resistance, represented by an elevated TyG index, predicts an increased risk of mortality in critically ill patients.

Notably, we revealed that the TyG index was not significantly related to the risk of mortality among DM patients. This phenomenon may be associated with various factors. In diabetic patients, insulin resistance is often a prominent physiological characteristic. Due to the inherent involvement of insulin resistance and glucose metabolism issues in diabetes, the variations in the TyG index may not be as pronounced in diabetic patients as in nondiabetic individuals. In such cases, the TyG index may lose its independent predictive value for mortality risk. Diabetic patients typically undergo medication treatments, including insulin and oral antidiabetic drugs, to maintain blood glucose levels. These treatments may influence changes in the TyG index and could also impact mortality risk. Treatment intervention may overshadow the independent contribution of the TyG index to mortality risk. Diabetic patients often experience various complications, such as cardiovascular diseases and kidney disorders. These complications may exert a direct and strong influence on mortality risk, rendering the TyG index relatively secondary. Additionally, there is limited research incorporating subgroup analyses, and further studies may be needed in the future to provide more clarity.

As reported by Cheng et al, the dynamic change in the TyG index might be superior to the effect of the baseline TyG index in predicting mortality risk among critically ill patients. The changing trend of the TyG index can dynamically reflect the patient’s metabolic status. In critically ill patients, metabolic status can be influenced by various factors, including disease progression and treatment outcomes. Monitoring the changing trend of the TyG index allows for a more comprehensive understanding of the patient’s metabolic dynamics, thereby enabling a more accurate prediction of the risk of mortality. The changing trend of the TyG index provides timely feedback on the development of the patient’s condition. Medical teams can adjust treatment strategies promptly based on the changing trend of the TyG index, adopting more effective measures to address metabolic disturbances and other complications. In addition, various influencing factors, including insulin resistance, glucose metabolism, and inflammatory status, can influence the trend of changes in the TyG index. This contributes to a more holistic assessment of the patient’s overall health, enhancing the accuracy of predicting the risk of mortality. There are individual differences in metabolic status among different patients, and the changing trend of the TyG index can better reflect these differences. By focusing on the changing trend, a more nuanced risk assessment can be conducted based on the individual’s metabolic characteristics, resulting in a more personalized and accurate prediction. Therefore, the association between dynamic changes in the TyG index and mortality risk should be further clarified in future relevant studies.

To date, this is the first meta-analysis to explore the relationship between the TyG index and mortality risk in critically ill patients, and our results have indicated their obvious association. In addition, to further enhance the clinical application of the TyG index among critically ill patients, we conducted subgroup analysis stratified by comorbidities such as DM and hypertension.

There are several limitations in this meta-analysis. First, all included studies were retrospective. Second, the classification methods and definitions of the TyG index in the included studies varied to some extent, which may have led to some biases. Third, due to the lack of original data, we were unable to conduct additional subgroup analyses based on other important parameters, such as disease type.

## 5. Conclusion

The TyG index is significantly associated with mortality among critically ill patients without DM, and an elevated TyG index predicts an increased risk of mortality.

## Author contributions

**Conceptualization:** Mengzhen Nie, Yu Xu.

**Formal analysis:** Mengzhen Nie, Biantong Jiang.

**Investigation:** Mengzhen Nie.

**Methodology:** Mengzhen Nie, Biantong Jiang.

**Writing – original draft:** Mengzhen Nie.

**Data curation:** Biantong Jiang.

**Writing – review & editing:** Biantong Jiang, Yu Xu.

**Supervision:** Yu Xu.

## Supplementary Material


