# Supplementary figures and images for: Association between the triglyceride–glucose index and mortality in critically ill patients: A meta-analysis
Source: Medicine (Baltimore). 2024 Aug 16;103(33):e39262. doi: 10.1097/MD.0000000000039262 (PMC11332756; doi:10.1097/MD.0000000000039262)

2024-2-2


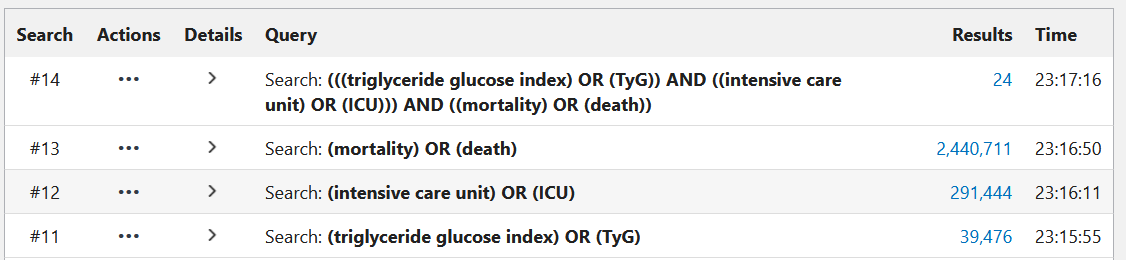

Supplement: Supplementary file 1 [file medi-103-e39262-s001.docx]
